# Stability of associations between neuroticism and microstructural asymmetry of the cingulum during late childhood and adolescence: Insights from a longitudinal study with up to 11 waves

**DOI:** 10.1002/hbm.26157

**Published:** 2022-11-25

**Authors:** Anna Plachti, William F. C. Baaré, Louise Baruël Johansen, Wesley K. Thompson, Hartwig R. Siebner, Kathrine Skak Madsen

**Affiliations:** ^1^ Danish Research Centre for Magnetic Resonance, Centre for Functional and Diagnostic Imaging and Research Copenhagen University Hospital ‐ Amager and Hvidovre Copenhagen Denmark; ^2^ Department of Radiology and Division of Biostatistics, Population Neuroscience and Genetics Lab University of California San Diego, San Diego School of Medicine La Jolla California USA; ^3^ Department of Neurology Copenhagen University Hospital ‐ Bispebjerg and Frederiksberg Copenhagen Denmark; ^4^ Institute for Clinical Medicine, Faculty of Medical and Health Sciences University of Copenhagen Copenhagen Denmark; ^5^ Radiography, Department of Technology University College Copenhagen Denmark

**Keywords:** adolescence, cingulum asymmetry, DTI, fractional anisotropy, neuroticism, sex differences

## Abstract

Adolescence is characterized by significant brain development and marks a period of the life span with an increased incidence of mood disorders, especially in females. The risk of developing mood disorders is also higher in individuals scoring high on neuroticism, a personality trait characterized by a tendency to experience negative and anxious emotions. We previously found in a cross‐sectional study that neuroticism is associated with microstructural left–right asymmetry of the fronto‐limbic white matter involved in emotional processing, with opposite effects in female and male adolescents. We now have extended this work collecting longitudinal data in 76 typically developing children and adolescents aged 7–18 years, including repeated MRI sampling up to 11 times. This enabled us, for the first time, to address the critical question, whether the association between neuroticism and frontal‐limbic white matter asymmetry *changes* or *remains stable* across late childhood and adolescence. Neuroticism was assessed up to four times and showed good intraindividual stability and did not significantly change with age. Conforming our cross‐sectional results, females scoring high on neuroticism displayed increased left–right cingulum fractional anisotropy (FA), while males showed decreased left–right cingulum FA asymmetry. Despite ongoing age‐related increases in FA in cingulum, the association between neuroticism and cingulum FA asymmetry was already expressed in females in late childhood and remained stable across adolescence. In males, the association appeared to become more prominent during adolescence. Future longitudinal studies need to cover an earlier age span to elucidate the time point at which the relationship between neuroticism and cingulum FA asymmetry arises.

## Graphical abstract

Microstructure of the cingulum was studied longitudinally with MRI during late childhood and adolescence. Higher neuroticism was related to left–right fractional anisotropy (FA) asymmetry of the cingulum in females and decreased asymmetry in males. The association in females was already present in late childhood and remained stable across adolescence.

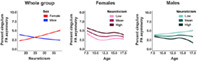

**Abbreviations**
AD: axial diffusivity
DT: diffusion tensor
DTI: diffusion tensor imaging
DWI: diffusion‐weighted imaging
FA: fractional anisotropy
MD: mean diffusivity
RD: radial diffusivity
ROI: region‐of‐interest
UF: uncinate fasciculus
vmPFC: ventromedial prefrontal cortex
vmPFC_WM_
: ventromedial prefrontal cortex white matter
WM: white matter

## 1 INTRODUCTION

Adolescence is associated with an increased incidence of neuropsychiatric disorders including mood disorders such as anxiety or depression (Bradley, 2001; Paus et al., 2008). The risk of developing mood disorders is increased in individuals scoring high on the personality trait neuroticism (Hettema et al., 2006; Kendler & Myers, 2010; Tully et al., 2015). Neuroticism reflects an individual's tendency toward experiencing negative emotions, such as anxiousness, sadness, worry, and difficulties coping with stress (Bienvenu et al., 2001). Neuroticism is moderately heritable (Sanchez‐Roige et al., 2018; van den Berg et al., 2014) and the association between neuroticism and anxiety or depressive symptoms and major depression seems largely to be due to common genetic risk factors (Kendler & Myers, 2010; Luciano et al., 2018). Moreover, females generally score higher on neuroticism than males throughout most of the lifespan (Chapman et al., 2007; Schmitt et al., 2017; Soto et al., 2011). This difference may already become apparent in adolescence around the age of 14 years (De Bolle et al., 2015), consonant with evidence that an equal female–male prevalence of anxiety and mood disorders before puberty changes to a 2:1 female–male prevalence after puberty (Goodwin & Gotlib, 2004; Paus et al., 2008). This raises the question, if observed sex differences in neuroticism in the peri‐pubertal period are associated with any brain changes.

Neuroimaging studies investigating the neural correlates of neuroticism and other negative emotionality traits such as harm avoidance have mostly been conducted in adult cohorts (Mincic, 2015; Opel et al., 2020; Privado et al., 2017; Servaas et al., 2013) and thus cannot shed light on whether any such neural correlates are already present in childhood and/or adolescence. Structural magnetic resonance imaging (MRI) studies have shown associations between higher neuroticism scores and a wide range of brain regions, including smaller total brain volume and smaller fronto‐temporal surface area (Bjørnebekk et al., 2013), thicker left inferior parietal cortex (Privado et al., 2017), larger left amygdala and anterior parahippocampal gyrus volumes and smaller grey matter volumes in anterior brain regions such as orbitofrontal and cingulate cortex with a predominance in the left hemisphere (Mincic, 2015). Interestingly, cingulotomy, that is, the surgical lesioning of the cingulum that has been used to reduce anxiety, depression and other psychiatric symptoms, have been observed to affect patients' personality by reducing mood‐related emotional tension and agitation (Cohen et al., 2001). Diffusion‐weighted imaging (DWI) studies have revealed associations of neuroticism and other negative emotionality‐related personality traits with global white matter tissue measures of fractional anisotropy (FA) and mean‐ and radial diffusivities (MD, RD) (Bjørnebekk et al., 2013), as well as with reduced FA in major white matter fiber tracts such as uncinate fasciculus and cingulum bundle (Mincic, 2015; Privado et al., 2017). Despite the distributed observed associations, many studies have implicated a fronto‐limbic network in negative emotionality traits, including the cingulum, uncinate fasciculus and the white matter underlying the ventromedial prefrontal cortex (vmPFC_WM_) (Cremers et al., 2010; Forbes et al., 2014; Gardiner et al., 2015; Madsen et al., 2012; Madsen et al., 2018; Mincic, 2015; Moshirian Farahi et al., 2019).

Recently, in a previous cross‐sectional study of the same cohort of children and adolescents, albeit smaller sample size and younger age range (10–15 years), than included in the present study, we found that neuroticism was associated with the relative balance between left and right hemispheric white matter tracts (i.e., asymmetry), rather than the absolute FA values of these white matter tracts (Madsen et al., 2018). These associations differed between sexes, with higher neuroticism scores being associated with decreased left relative to right cingulum FA in males, while in females, higher neuroticism scores were related to increased left relative to right cingulum and ventromedial prefrontal white matter FA. Our finding in adolescent males aligned well with our similar observation in a cohort of healthy adults aged 19–86 years that contained approximately twice as many males as females (Madsen et al., 2012). Furthermore, the observed associations in children and adolescents became stronger with increasing age in males but not in females (Madsen et al., 2018). Although intriguing, the latter needs to be scrutinized using a longitudinal design. Notably, the cingulum and uncinate fasciculus undergo continuous maturation, that is, age‐related increases in FA and decreases in MD, throughout childhood and adolescence and into early adulthood (Lebel & Beaulieu, 2011). Moreover, the relative balance between left and right cingulum FA was previously reported to be either symmetrical except for the anterior part (Thiebaut de Schotten et al., 2011), or displaying a left‐to‐right asymmetry not related to sex (Gong et al., 2005; Madsen et al., 2012; Takao et al., 2013). Generally, interhemispheric and regional brain asymmetries are common in the human brain and partly cause or facilitate functional specializations (i.e., handedness, language), which may already be evident during fetal gestation (Francks, 2015; Hepper, 2013). Hemispheric asymmetry reported in the electroencephalography (EEG) literature has previously been related to approach/withdrawal behavior and emotional processing (Grimshaw & Carmel, 2014; Nusslock et al., 2015). Furthermore, sex appears to be a crucial variable in emotional processing and lateralized regional activity, with negative images evoking stronger EEG activity in the left hemisphere in women than in men and stronger right hemisphere activity in men than in women (Gasbarri et al., 2007). Moreover, functional MRI (fMRI) and positron emission tomography (PET) activity related to memory for emotional stimuli was associated to right but not left amygdala activity in men and oppositely, left but not right amygdala activity in women (Cahill et al., 2001, 2004; Canli et al., 2002). Finally, whereas sex differences in associations between neuroticism and brain structure may already be present in adolescence (Blankstein et al., 2009; Madsen et al., 2018), the possible effect of sex on negative emotionality‐related traits and brain structure has not been systematically investigated yet and findings are generally inconclusive (Avinun et al., 2020; Mincic, 2015; Nostro et al., 2017).

In the present longitudinal study, we aimed to elucidate sex differences in fronto‐limbic white matter correlates of neuroticism and to characterize how the relationship between neuroticism and fronto‐limbic white matter might change with age across childhood and adolescence. To this end, we investigated typically developing children and adolescents aged 7 and 18 years, who underwent DWI up to 11 times and were assessed on neuroticism up to four times. Based on our cross‐sectional findings in the same sample (Madsen et al., 2018), we expected that our longitudinal data would affirm the previously observed cross‐sectional associations between neuroticism and cingulum and vmPFC_WM_ FA asymmetries. Specifically, for our first hypothesis, we expected that higher neuroticism scores would be associated with increased left relative to right cingulum and vmPFC_WM_ FA asymmetries in females and to decreased left relative to right cingulum FA asymmetry in males. Furthermore, in our second hypothesis, we expected that, the neuroticism associations with cingulum and vmPFC_WM_ FA asymmetry would differ as a function of age within the investigated age range.

## 2 MATERIALS AND METHODS

### 2.1 Study design and participants

The present longitudinal study included data from 76 typically developing children and adolescents (47 females, 29 males) aged 7.5–18.9 years (mean = 12.4, standard deviation [SD] = 2.4), who were enrolled in the longitudinal HUBU (“*Hjernens Udvikling hos Børn og Unge*”) study. The HUBU study was initiated in 2007, where 95 participants (55 females, 40 males) aged 7–13 years and their families were recruited from three elementary schools in the Copenhagen suburban area. All children whose families volunteered were included, except for children who according to parent‐reported screening questionnaires had any known history of neurological or psychiatric disorders or significant brain injury. Prior to participation, children and their parents were informed about the aims and procedures of the study in oral and written language and in accordance with children's assent, all parents gave their informed written consent. Further, informed written consent was obtained from the participants themselves when they turned 18 years. The study was approved by the Ethical Committees of the Capital Region of Denmark (H‐KF‐01‐131/03 and H‐3‐2013‐037) and performed in accordance with the Declaration of Helsinki. Participants were assessed up to 13 times with 6 months intervals between the first 10 assessments, 1‐year interval for the 11th assessment, and 3‐year intervals for Assessments 12 and 13.

**FIGURE 1:**
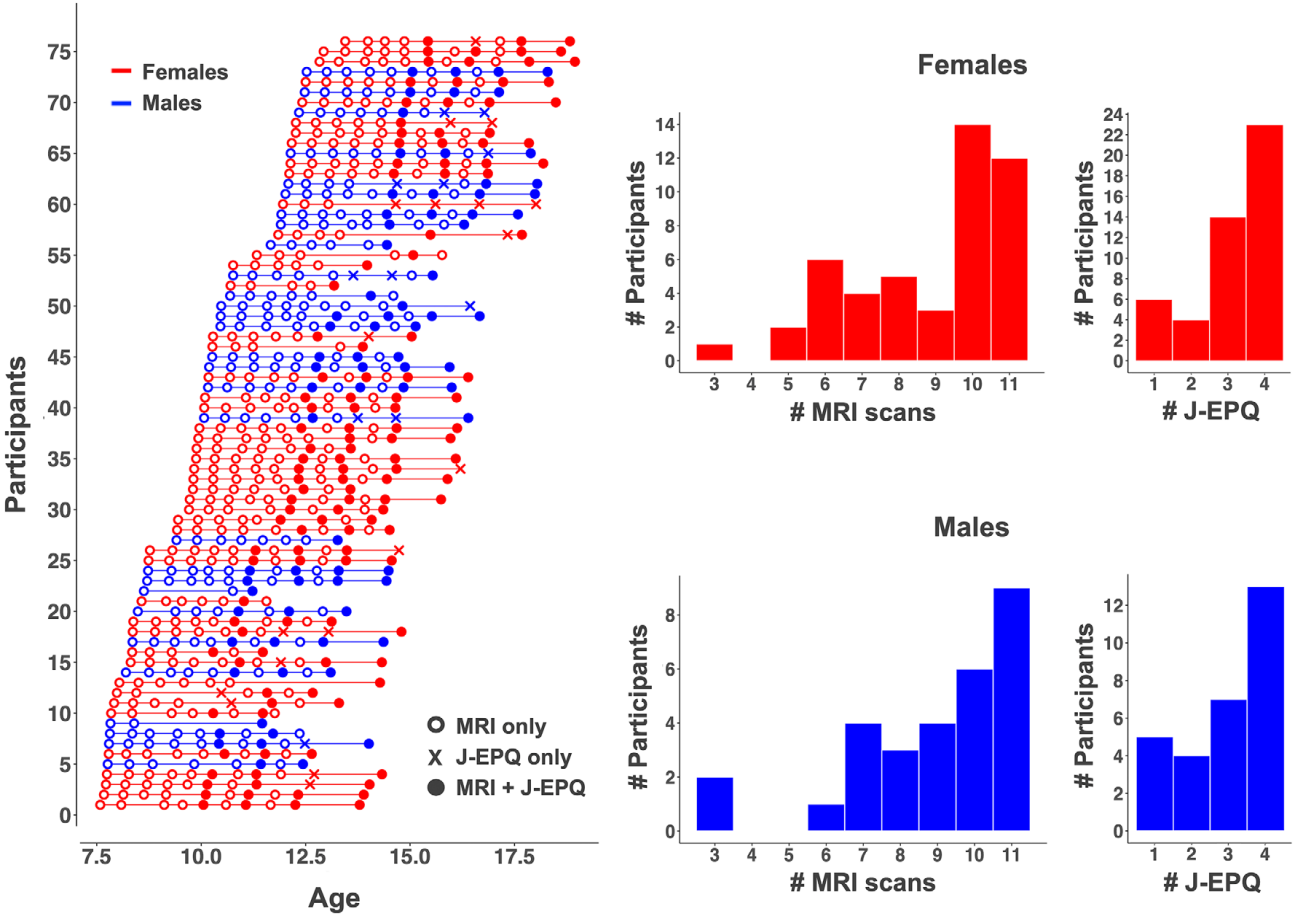
Left: Number of longitudinal observations and age at each assessment for each of the 76 participants. Assessments including MRI and the Junior Eysenck Personality Questionnaire (J‐EPQ, in the present study only neuroticism scores were used—see text) are shown as closed circles, MRI only as open circles, and J‐EPQ only as “x.” Right: Histograms depicting the number of participants that have a certain number of MRI scans or J‐EPQ assessments, for males and females separately. Females (*n* = 47) are shown in red, and males (*n* = 29) in blue. # = “number of”

The present study included data from the first 11 assessments of the HUBU study. A total of 19 participants were excluded from further analyses, due to lack of acquired neuroticism data (*n* = 16), receiving a psychiatric diagnosis after study initiation (*n* = 2), or an incidental clinical finding on the MRI scan (*n* = 1). Our final sample consisted of 76 participants (47 females and 29 males) aged 7.5–18.9 years (mean = 12.4, standard deviation = 2.4). From these, we excluded 64 MRI sessions, because the participant did not finish the MRI scanning session (1 participant, 2 assessments), was not scanned due to metallic dental braces (13 participants, 30 assessments), had poor MR‐image quality due to, for example, excessive motion, slice dropouts, spikes or radiofrequency interference artifacts (17 participants, 23 assessments), had acquired a brain injury after baseline (1 participant, 8 assessments) or the assessment was accidently left out from the preprocessing (1 assessment). A total of 675 valid observations (range = 3–11, mean = 8.9 observations per subject) were included in the statistical analyses. A summary of the longitudinal assessments and participants' age at each assessment is presented in Figure 1. According to the Edinburg Handedness Inventory (EHI), 68 participants were right‐handed (EHI score ≥ 40) and 8 were left‐handed (EHI score ≤ −40). Highest level of maternal and paternal education was transformed into years of education using national norms and used to estimate average years of parental education for all participants (mean = 13.8, SD = 2.0). Data from the HUBU cohort have previously been used in cross‐sectional (Angstmann et al., 2016; Gonzalez et al., 2021; Klarborg et al., 2013; Madsen et al., 2010, 2011, 2018; Vestergaard et al., 2011) and longitudinal (Madsen et al., 2020) studies examining brain–behavioral relationships.

### 2.2 Personality assessment

Participants completed an adapted Danish version of the 81‐item Junior Eysenck Personality Questionnaire (J‐EPQ) (Eysenck & Eysenck, 1975; Nyborg et al., 1982) on the same day as the MR‐scanning in rounds 6, 8, 10, and 11. The J‐EPQ measures three major dimensions of personality, that is, neuroticism, extraversion, and psychoticism. Individual J‐EPQ statements were read out loud by the test administrator and participants subsequently rated how well each statement fitted them. In HUBU, we extended the original “Yes” or “No” rating scale to: Strongly agree/Agree/Disagree/Strongly disagree, to allow for a more fine‐grained evaluation of the personality traits. The answers were scored on a 0‐3‐point scale, with 0 reflecting strongly disagree. In the present study, we focused on the neuroticism scale that consists of 20 items. The neuroticism scale showed good‐to‐excellent internal consistency using either the 2‐point or 4‐point rating scales (Madsen et al., 2018). Our 2‐point scores on neuroticism in males and females were in line with previous reported Danish norm scores (Nyborg et al., 1982).

### 2.3 Image acquisition

“All subjects were scanned using a 3T Siemens Magnetom Trio MR scanner (Siemens, Erlangen, Germany) with an eight‐channel head coil (Invivo, FL, USA). All acquired scans were aligned parallel to the anterior commissure–posterior commissure line. T1‐weighted images of the whole head were acquired using a 3D MPRAGE sequence (TR = 1550 ms, TE = 3.04 ms, matrix 256 × 256, 192 sagittal slices, 1 × 1 × 1 mm^3^ voxels, acquisition time = 6:38). T2‐weighted images of the whole head were acquired using a 3D turbo spin echo sequence (TR = 3000 ms, TE = 354 ms, FOV = 282 × 216, matrix = 256 × 196, 192 sagittal slices, 1.1 × 1.1 × 1.1 mm^3^ voxels, acquisition time = 8:29). Whole brain diffusion‐weighted (DW) images were acquired using a twice‐refocused balanced spin echo sequence that minimized eddy current distortion (Reese et al., 2003). Ten non‐DW images (*b* = 0) and 61 DW images (*b* = 1200 s/mm^2^), encoded along independent collinear diffusion gradient orientations, were acquired (TR = 8200 ms, TE = 100 ms, FOV = 220 × 220, matrix = 96 × 96, GRAPPA: factor = 2, 48 lines, 61 transverse slices with no gap, 2.3 × 2.3 × 2.3 mm^3^ voxels, acquisition time = 9:50). A gradient echo field map was acquired to correct B_0_ field distortions (TR = 530 ms, TE[1] = 5.19 ms and TE[2] = 7.65 ms, FOV = 256 × 256; matrix = 128 × 128, 47 transverse slices with no gap, voxel size = 2 × 2 × 3 mm^3^, acquisition time = 2:18).” (Madsen et al., 2018).

### 2.4 Image evaluation

An experienced neuroradiologist evaluated all baseline MRI scans, and all, but one, were deemed without significant clinical pathology. Prior to analysis and blind to behavioral data, all raw MR‐images were visually inspected to ascertain image quality and excluded if not of sufficient quality (see Section 2.1).

### 2.5 Construction of the diffusion tensor images

The preprocessing of MRI images is identical as described in the study by Madsen et al. (2020) and subsequently directly quoted here.

“Image preprocessing was done using MATLAB scripts that were mainly based on SPM 8 routines (Wellcome Department of Cognitive Neurology, University College London, UK). The T1‐ and T2‐weighted images were rigidly oriented to MNI space (six‐parameter mutual information) and corrected for spatial distortions due to nonlinearity in the gradient system of the scanner (Jovicich et al., 2006) (note that at this point no reslicing was performed). T2‐weighted images were then rigidly co‐registered to the T1‐weighted image. To align the DWI images to the T1‐weighted image, the mean b0 image was first rigidly registered to the T2‐weighted image, after which all DW images were co‐registered to the mean b0 image (no reslicing). Next, all co‐registered DWI images were corrected for spatial distortions using a voxel displacement map based on the acquired b0 field map (Andersson et al., 2001) and the scanner‐specific gradient non‐linearity profile (Jovicich et al., 2006). Subsequently, all images were resliced using tri‐linear interpolation. Importantly, the above procedure ensures that only one re‐slicing step was employed. Diffusion gradient orientations were adjusted to account for any applied rotations. The least‐squares‐fit by non‐linear optimization, employing a Levenburg‐Marquardt algorithm and constrained to be positive definite by fitting its Cholesky decomposition, implemented in Camino was used to fit the diffusion tensor (DT) (Jones & Basser, 2004). Finally, for each subject we estimated movement during DWI scanning by calculating the root mean square deviation (RMS) of the six rigid body transformation parameters resulting from co‐registering DWI images to the mean b0 image (Jenkinson & Smith, 2001; Taylor et al., 2016), using a spherical volume radius of 60 mm to approximate the brain.” (Madsen et al., 2020)

### 2.6 Spatial normalization of the longitudinal diffusion tensor images

“The diffusion tensor images were spatially normalized using DTI‐TK, which uses the high dimensional information of the diffusion tensor to achieve highly accurate normalizations (Zhang et al., 2007). We employed an unbiased longitudinal two‐step approach (Keihaninejad et al., 2013). First, within each subject all DT images over all time points were registered together to create within‐subject DT image templates. Secondly, within‐subject template DT images were registered together to create a between‐subject DT template image. Concatenation of the within‐ and between subject registration deformation fields was used to warp individual DWI volumes into a common study specific space. Fractional anisotropy (FA), axial diffusivity (AD = λ1) and radial diffusivity (RD = (λ2 + λ3)/2) images were created. Finally, non‐brain voxels in FA and diffusivity images were removed by employing a brain mask based on warped b0 images” (Madsen et al., 2020). All normalized images were visually inspected to ascertain that they had been correctly normalized to both the within‐subject template as well as the between‐subject template.

### 2.7 Tract‐based spatial statistics

Tract‐Based Spatial Statistics (TBSS) (Smith et al., 2006), part of FSL 5.0.9, was used to create a mean FA skeleton, representing the centers of all tracts common to the group. Instead of using the standard TBSS normalization steps, we first aligned the between‐subject FA template image, derived from DTI‐TK, to 1 mm^3^ MNI space by means of a 12 parameter affine registration using flirt (FSL) to FSL's modified version (asymmetric) of the MNI ICBM 152 nonlinear sixth Generation Symmetric Average Brain Stereotaxic Registration Model (Grabner et al., 2006). Subsequently, we applied the resulting transformation matrix to the between‐subject diffusivity images and the normalized individual DT images. “Next, the between‐subject FA template image was entered into the TBSS processing stream using the ‘tbss_skeleton’ script, part of the ‘tbss_3_postreg’ processing step, in which the between‐subject FA template image was thinned to create a mean FA skeleton. The mean FA skeleton was thresholded at FA > 0.2 and contained 102,983 1 mm^3^ interpolated isotopic voxels, corresponding to approximately 22% of the voxels (in the mean FA map across subjects and time points) with FA above 0.2.” (Madsen et al., 2020). Next, all participants' aligned FA images were projected onto the mean FA skeleton by locating the voxels with the highest local FA value perpendicular to the skeleton tracts and assigning these values to the skeleton. Finally, the skeleton projections were applied on the AD and RD data.

### 2.8 Regions‐of‐interest

**FIGURE 2:**
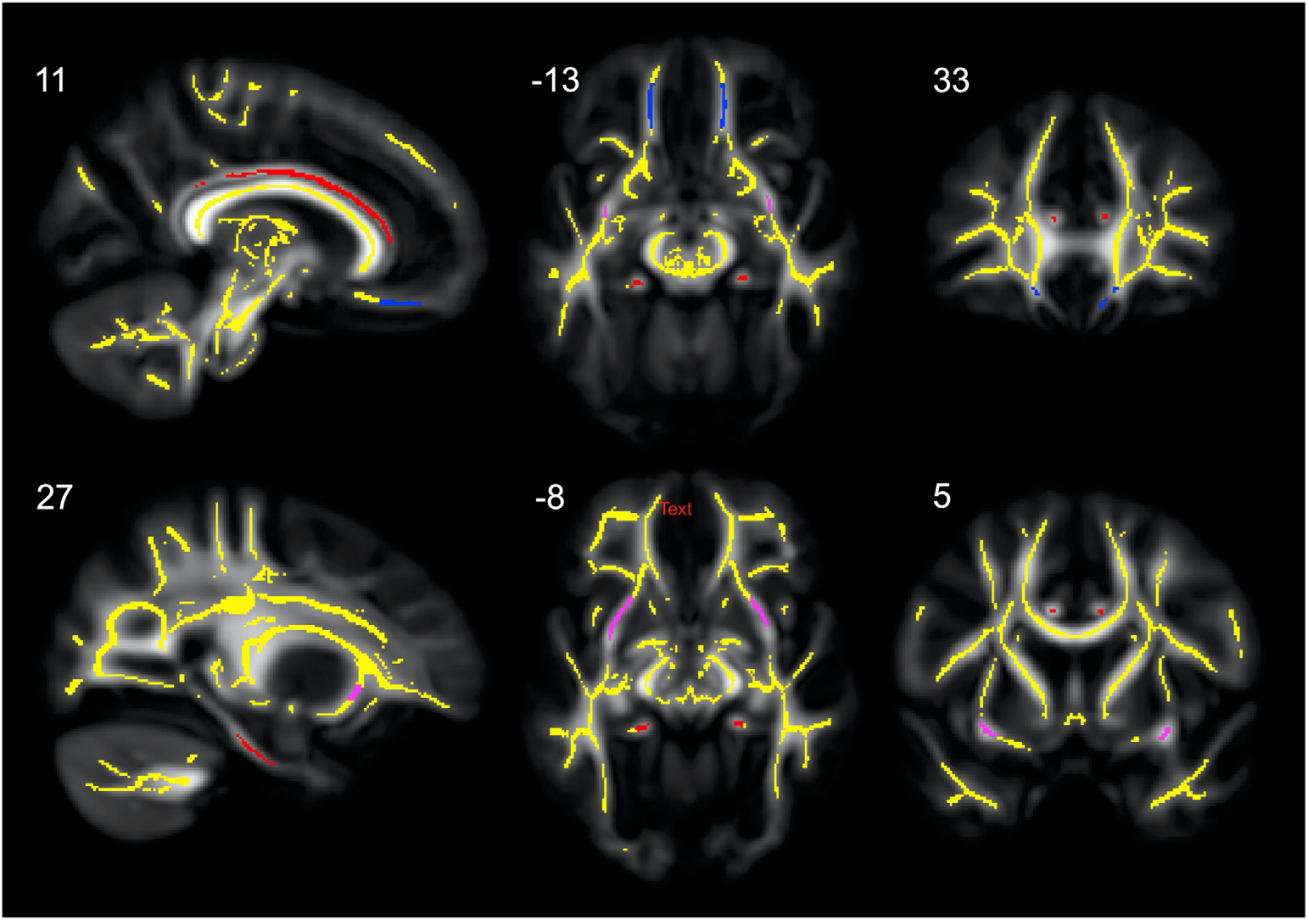
Regions‐of‐interest (ROIs) used in the cingulum bundle (red), uncinate fasciculus (UF, magenta) and the white matter underlying the ventromedial prefrontal cortex (vmPFCWM, blue) overlaid on the TBSS skeleton (yellow) and the mean fractional anisotropy (FA) map. ROIs are shown in sagittal (*x*), coronal (*y*), and axial (*z*) views with the corresponding MNI coordinates given above each slice.

We extracted FA, AD, and RD values from left‐ and right‐sided regions‐of‐interest (ROIs) to test specific hypotheses and to determine the anatomical specificity of observed associations. White matter ROIs included the cingulum, the uncinate fasciculus (UF) and the white matter underlying vmPFC. The ROIs were drawn manually onto the mean skeleton overlaid on the mean FA image using FSLview. We chose to manually delineate our ROIs based on anatomical information instead of using existing atlas ROIs to get a better fit, since affine registration of our child/adolescent cohort template to the MNI template does not ensure accurate registration of individual fiber tracts. The ROIs are shown in Figure 2. The cingulum ROIs included all skeleton segments within the cingulum and excluded any segments intersecting but diverging from the main body of the cingulum. The skeleton segments representing the cingulum were clearly distinguishable from all other skeleton segments. The left and right cingulum ROIs contained 643 and 622 voxels, respectively. The vmPFC_WM_ ROIs included the skeleton segments in the white matter underlying the left and right vmPFC, while excluding segments in the frontal pole. The most anterior part of the cingulate sulcus and corpus callosum were used as landmarks to define, respectively, the anterior and posterior borders of the vmPFC ROIs. The vmPFC_WM_ ROIs extended between MNI‐coordinates *y* = 51 to *y* = 33, both included. The left and right vmPFC_WM_ ROIs contained 142 and 162 voxels, respectively. The UF ROIs were delineated using anatomical information in the color‐coded mean FA map in which red, green, and blue, respectively, represent left–right, anterior–posterior, and superior–inferior directions. The UF was defined in accordance with an MRI atlas of the human white matter (Mori et al., 2005), and additional guidance was provided by the probabilistic UF in the JHU White‐Matter Tractography Atlas (Hua et al., 2008), implemented in FSLview, thresholded at 0.2. The ROIs included central UF segments in the external/extreme capsule and the stem of the temporal lobe (respectively, green and blue in the color‐coded FA map), while skeleton segments extending toward the inferior frontal gyrus, the orbitofrontal cortex and the temporal pole were excluded. The left and right UF ROIs included 335 and 356 voxels, respectively. Finally, to test the anatomical specificity of observed associations, left (50,795 voxels) and right (51,843 voxels) hemispheric ROIs, including all skeleton segments within each hemisphere, were delineated using the mid‐sagittal plane (not included in either of hemispheric ROIs).

### 2.9 Left–right asymmetry measures

Left–right ROI asymmetries were calculated for the different DTI parameters as the difference between left and right ROI values expressed as a percentage of the bilateral mean, with positive values indicating larger left relative to right measures:

$${\left(\left(2^{*}\;\left(\text{left}–\text{right}\right)\right)/\left(\text{left}+\text{right}\right)\right)}^{*}\;100$$

### 2.10 Statistical analyses

Statistical analyses were performed in RStudio 3.6.2 (R Core Team, 2019). The longitudinal analyses were conducted with generalized additive mixed models (GAMM) using the mgcv package, version 1.8‐31 and the nlme package, version 3.1‐142 (Pinheiro et al., 2013). GAMM easily handles unbalanced longitudinal data, such as unequal number of timepoints and/or unequal intervals between timepoints and uses nonlinear smooth functions to fit complex longitudinal data without a priori assumptions of the shape of the trajectories. The *estimated degrees of freedom* (edf) denote the degrees of freedom used by the smooth function to fit the data. An edf value of 1 represents a linear relationship with a straight regression line, whereas increasingly larger edf values reflect increasingly more nonlinear relationships.

Dependent variables were the ROI DTI or ROI DTI asymmetry measures, for example, ROI FA or ROI FA asymmetry. Sex was input as a covariate, coded with females = 1 and males = 0. The smooth terms were used for, respectively, age and age‐by‐sex interaction effects. Participant and within‐subject age were used as random effects to fit individual intercepts and slopes, respectively. We used the default thin plate regression splines smoothing function to fit the data with default penalizing parameters. Further, we used five knots (k), representing the k‐1 number of basis functions used to generate the smooth function, as we previously have shown that this reduces the risk of overfitting (Madsen et al., 2020). The smoothing parameters were estimated with the restricted maximum likelihood method (REML). All our continuous variables, that is, age, neuroticism scores, parental education, RMS, and ROI DTI measures, were z‐transformed.

#### 2.10.1 Age, sex, and age‐by‐sex effects on longitudinal neuroticism scores

Prior to testing our hypothesis, we assessed the intraindividual reliability or agreement of the longitudinal neuroticism scores using the intraclass‐correlation coefficient (ICC) approach (two‐way mixed effects model) (Koo & Li, 2016). Moreover, we tested for age, sex, and age‐by‐sex interaction effects on the longitudinal neuroticism data (neuroticism_long_) (Model 1) and possible additional handedness and parental education effects (Model 2) to assess whether neuroticism changed with age across childhood and adolescence and was associated with the additional covariates.

Model 1 Neuroticism_long_ = age + sex + age‐by‐sex.

Model 2 Neuroticism_long_ = age + sex + age‐by‐sex + parental education + handedness.

#### 2.10.2 Age, sex, and age‐by‐sex and subject movement effects on longitudinal ROI FA measures

Next, we assessed the longitudinal maturational trajectories of ROI FA with GAMM by testing for age, sex, age‐by‐sex, and RMS effects on left or right ROI FA, or ROI FA asymmetry (Model 3) and possible additional handedness and parental education effects (Model 4).

Model 3 ROI FA or FA asymmetry = age + sex + age‐by‐sex + RMS.

Model 4 ROI FA or FA asymmetry = age + sex + age‐by‐sex + RMS + parental education + handedness.

#### 2.10.3 Testing of a priori hypotheses: Associations between ROI FA asymmetry and neuroticism

**FIGURE 3:**
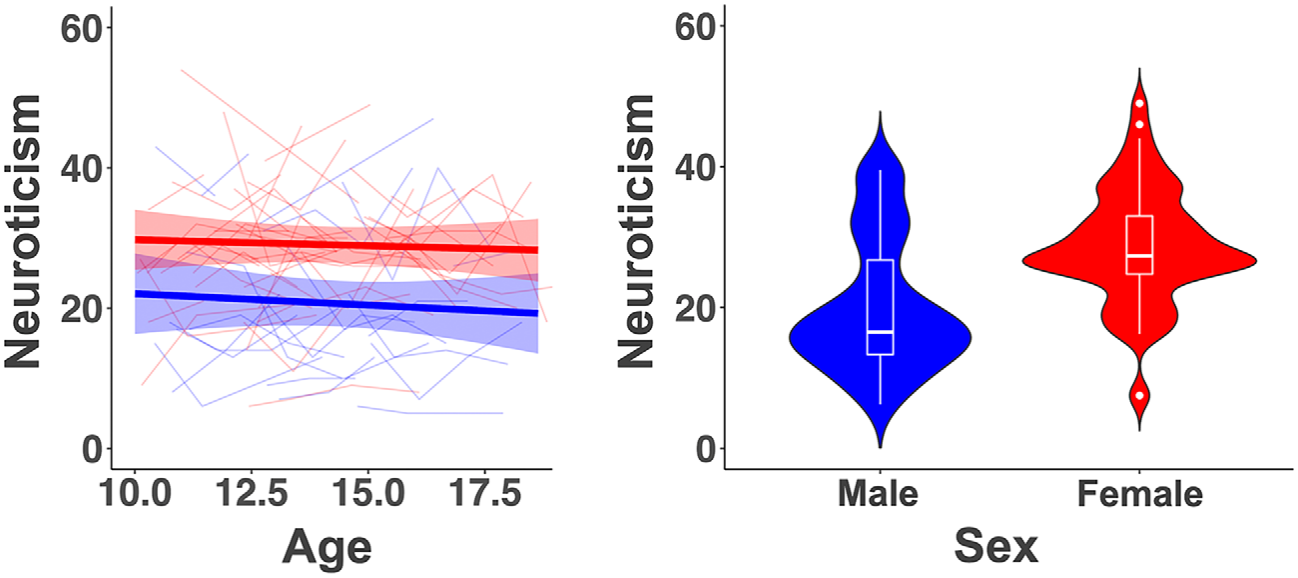
Left: Spaghetti plot of neuroticism_long_ overlaid with GAMM estimated age trajectories with shaded 95% confidence intervals for males (blue, *n* = 29) and females (red, *n* = 47). Right: Violin and box plots showing neuroticism_mean_ scores for females and males. Females had significant higher neuroticism_mean_ scores than males

Since the ICC analysis revealed moderate‐to‐good reliability and excellent agreement of the intraindividual longitudinal neuroticism scores (see Section 3.1.1), and the neuroticism_long_ scores did not significantly change with age across childhood and adolescence (see Table 1 and Figure 3), we used the intraindividual mean neuroticism scores (neuroticism_mean_) when testing our hypotheses. This allowed us to include MRI scans from all time points. To test our first hypothesis, neuroticism_mean_ and neuroticism_mean_‐by‐sex were included as independent variables of interest, while controlling for age, sex, age‐by‐sex and RMS (Model 5a). In Model 5b, we tested our second hypothesis by also including neuroticism_mean_‐by‐age and neuroticism_mean_‐by‐age‐by‐sex interaction effects. Dependent variables of interest were cingulum or vmPFC_WM_ FA asymmetry. UF FA asymmetry was tested exploratively.

Model 5a ROI FA asymmetry = age + sex + age‐by‐sex + RMS + neuroticism_mean_ + neuroticism_mean_‐by‐sex.

Model 5b ROI FA asymmetry = age + sex + age‐by‐sex + RMS + neuroticism_mean_ + neuroticism_mean_‐by‐sex + neuroticism_mean_‐by‐age + neuroticism_mean_‐by‐sex‐by‐age.

**TABLE 1:** Age, sex, age‐by‐sex, and RMS on ROI FA, ROI FA asymmetry and neuroticism_long_

			Age			Sex		Age‐by‐sex			RMS	
Measures		Model	edf	*F*	*p*	*t*	*p*	edf	*F*	*p*	*t*	*p*
Neuroticism_long_		1	1.01	0.35	.554	4.00	**<10** ^**−5**^	1.00	0.05	.822		
Cingulum FA	Right	3	1.00	161.66	**<10** ^**−16**^	−1.69	.091	2.91	3.33	**.018**	−2.62	**.009**
	Left	3	1.12	149.73	**<10** ^**−16**^	−1.66	.098	3.01	3.49	**.016**	−0.62	.536
UF FA	Right	3	1.00	105.16	**<10** ^**−16**^	−0.71	.481	3.53	17.69	**<10** ^**−12**^	−4.08	**<10** ^**−5**^
	Left	3	1.00	220.30	**<10** ^**−16**^	−0.51	.609	2.86	10.70	**<10** ^**−6**^	−1.90	.058
VmPFC_WM_ FA	Right	3	1.00	12.07	**.001**	−0.84	.400	2.09	0.78	.563	−2.12	**.034**
	Left	3	2.61	13.34	**<10** ^**−18**^	−0.37	.709	2.32	1.45	.209	−2.13	**.034**
Cingulum FA	Asymmetry	3	1.12	1.12	.276	0.22	.826	3.09	2.18	.066	1.98	**.049**
UF FA	Asymmetry	3	2.83	6.82	**<10** ^**−3**^	0.13	.898	1.02	7.31	**.007**	1.61	.109
VmPFC_WM_ FA	Asymmetry	3	1.55	2.65	.18	0.38	.703	1.00	0.00	.951	−0.53	.596

*Note*: Each row represents a separate GAMM model predicting neuroticism_mean_ or ROI FA measures. The results for neuroticism_long_ are based on Model 1, while the results for ROI or ROI asymmetry are based on Model 3 (Section 2.10.2). *p* values below .05 (uncorrected) are shown in bold. Movement during scanning (RMS) was only included for brain measures.

Abbreviations: edf, estimated degrees of freedom; FA, fractional anisotropy; GAMM, generalized additive mixed model; RMS, root mean square movement; ROI, region‐of‐interest; UF, uncinate fasciculus; vmPFC, ventromedial prefrontal cortex; WM, white matter.

Since we were a priori interested in any association including neuroticism_mean_ (main and interaction effects), we applied a Bonferroni correction. For Model 5a, we had four associations of interest (two for cingulum and two for vmPFC FA asymmetry). Thus, a *p* value below .0125 was considered significant. Model 5b was corrected for eight associations of interest including neuroticism (four for cingulum and four for vmPFC FA asymmetry). Thus, a *p* value below .006 was considered significant.

In our exploratory analyses of UF FA asymmetry, in Model 5a, a Bonferroni‐corrected *p* value below .005 (corrected for 10 associations of interest, i.e., the eight a priori hypothesized, and the two exploratory ones for UF FA asymmetry) was considered significant. For Model 5b, a Bonferroni‐corrected *p* value below .004 (corrected for 12 associations of interest, i.e., the eight a priori hypothesized, and the four exploratory ones for UF FA asymmetry) was considered significant.

#### 2.10.4 Sex‐specific analyses

Contingent on observing significant neuroticism_mean_‐by‐sex effects on ROI FA asymmetry in Model 5, follow‐up analyses were conducted in females and males separately to further investigate the neuroticism_mean_‐by‐sex effects (Model 6) as well as to investigate whether any relationship between neuroticism_mean_ and ROI FA asymmetry in females or males changed with age (Model 7).

Model 6 ROI FA asymmetry = age + RMS + neuroticism_mean_.

Model 7 ROI FA asymmetry = age + RMS + neuroticism_mean_ + neuroticism_mean_‐by‐age.

#### 2.10.5 Follow‐up analyses

Contingent on observing significant neuroticism_mean_ or neuroticism_mean_‐by‐age effects on ROI FA asymmetry for males and females separately, we performed several planned follow‐up analyses. As no significant neuroticism_mean_‐by‐age effects on ROI FA asymmetry were observed (see Section 3.2.1), the follow‐up models were all extensions of Model 6. First, we assessed the anatomical specificity of the observed neuroticism_mean_ and ROI FA asymmetry association by including hemispheric FA asymmetry as an additional covariate. Second, we tested whether the observed association remained when including either with total brain volume (extracted from the longitudinal FreeSurfer pipeline; see Fuhrmann et al., 2022) or parental education and handedness as additional covariates. Third, we assessed the contribution of absolute right and left ROI FA values to neuroticism_mean_ by analyzing them separately. Finally, to further explore the nature of our observed FA findings, we performed similar analyses as for FA on ROI AD and ROI RD asymmetries.

#### 2.10.6 Effect size maps

We generated effect size maps to provide further information about possible associations between neuroticism_mean_ and FA values across the white matter skeleton. The effect size maps display the distribution of uncorrected *t*‐values of the associations between neuroticism_mean_ and FA in males and females separately, controlling for age and RMS (Model 6). The unthresholded *t*‐maps have been uploaded to NeuroVault.org (Gorgolewski et al., 2015) and are available at https://neurovault.org/collections/LRMGYBHV/.

## 3 RESULTS

### 3.1 Descriptive statistics

#### 3.1.1 Intraindividual reliability and agreement of the longitudinal neuroticism scores

The ICC analysis revealed a moderately to good intraindividual reliability (ICC = 0.74, 95% CI: 0.68–0.80) and the overall agreement achieved an excellent rating (ICC = 0.96, 95% CI: 0.95–0.97), indicating that neuroticism_long_ showed good intraindividual consistency across time during childhood and adolescence.

#### 3.1.2 Age, sex, age‐by‐sex, and RMS effects on neuroticism scores

We found no significant age or age‐by‐sex effects on the neuroticism_long_ scores (Table 1 and Figure 3), suggesting that neuroticism_long_ scores did not change significantly across childhood and adolescence. However, as expected, we observed a significant effect of sex with females scoring higher than males on neuroticism_long_ (*t* = 4.677, *p* < 10^−5^) and neuroticism_mean_ (Kruskal–Wallis *t*‐test: df = 1, C^2^ = 127.47, *p* < 10^−16^; Figure 3). Furthermore, higher neuroticism_long_ scores were significantly associated with lower parental education (*t* = −2.76, *p* = .006). No significant association was observed between neuroticism_long_ and handedness (*t* = −1.583, *p* = .115). Since the intraindividual reliability and agreement of neuroticism_long_ were good, and there were no significant age or age‐by‐sex effects on neuroticism_long_, we used the neuroticism_mean_ score of each individual for further analysis. In case a participant had only one neuroticism score (five males and six females), this score was used as the participant's “mean neuroticism score.” Neuroticism_mean_ was not significantly correlated with RMS (*r* = .035, *p* = .37; RMS median = 0.84, SD = 0.20, range: 0.5–2.9).

#### 3.1.3 Age, sex, age‐by‐sex, and RMS effects on ROI FA and FA asymmetry

**FIGURE 4:**
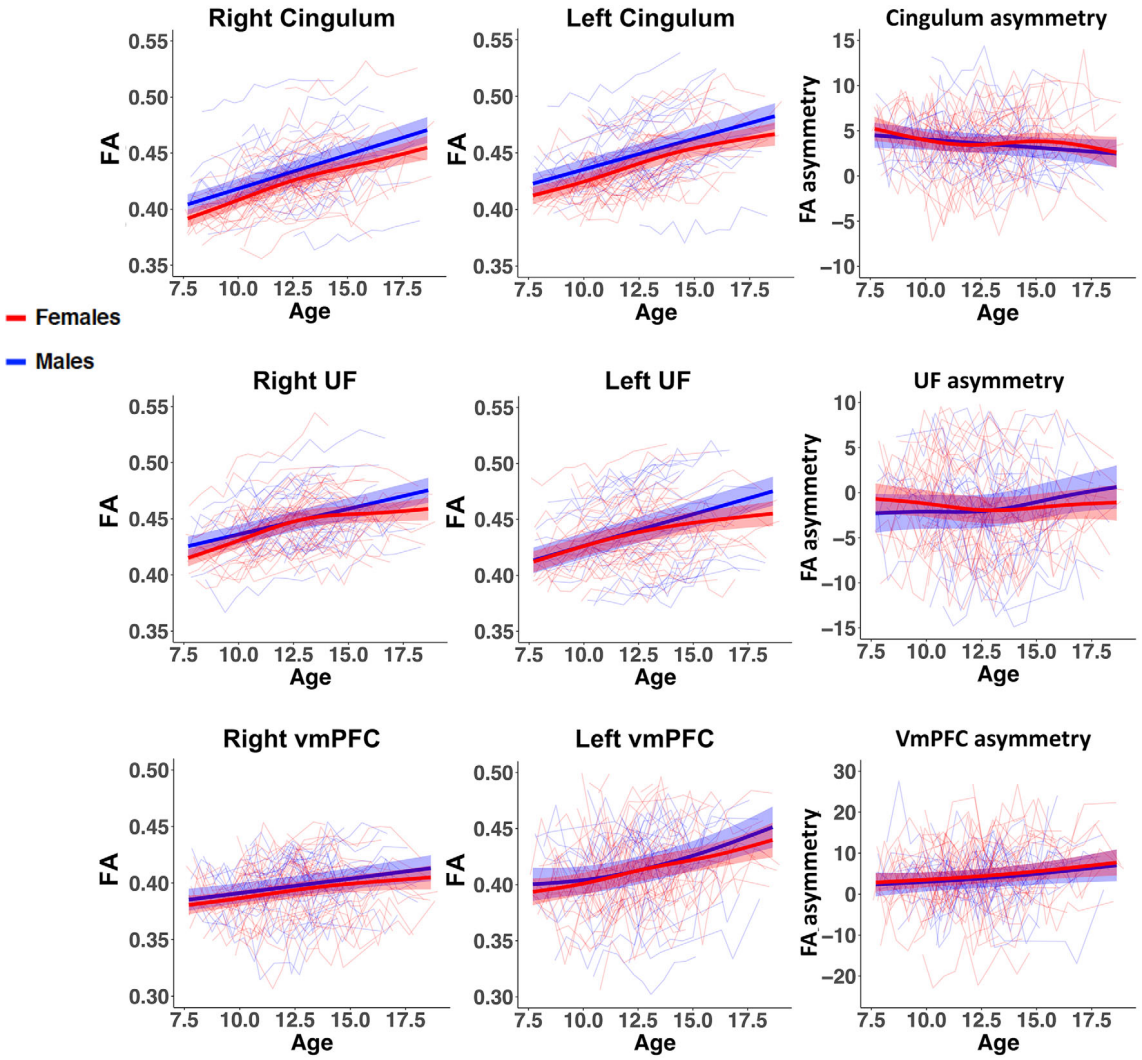
Spaghetti plots of cingulum, uncinate fasciculus (UF) and white matter underlying the ventromedial prefrontal cortex (vmPFCWM) fractional anisotropy (FA) overlaid with GAMM estimated age trajectories with shaded 95% confidence intervals for males (blue) and females (red)

Effects of age, sex, age‐by‐sex, and RMS on the ROI FA measures are reported in Table 1. Spaghetti plots overlaid with GAMM estimated age trajectories for males and females are shown in Figure 4. FA significantly increased with age in all right and left ROIs. Moreover, significant age‐by‐sex effects were found for right and left cingulum and UF FA, with males displaying more linearly increasing maturational trajectories, while females displayed more curvilinear trajectories that started to level off within the second half of the investigated age range. For ROI FA asymmetry, only the UF exhibited significant age and age‐by‐sex effects, with the UF changing from rightward FA asymmetry to less asymmetric FA with age in males, and slightly increased rightward FA asymmetry with age in females. In contrast, cingulum and vmPFC_WM_ FA asymmetries were relatively stable across this age range. No significant main effects of sex were observed for any of the ROI FA measures. Significant effects of movement (RMS) were found for several ROIs FA measures (Table 1). Moreover, RMS was significantly dependent on age (edf = 3.24, *F* = 20.97, *p* < .001), but not on sex (*t* = −0.552, *p* = .58) or age‐by‐sex (edf = 1.00, *F* = 0.67, *p* = .414). To account for potential movement effects on the DTI parameters, we included RMS in all our models.

When additionally controlling for parental education and handedness, observed age, age‐by‐sex and RMS effects remained, and a modest sex effect appeared, with males having higher left cingulum FA than females (*t* = −2.025, *p* = .043). Parental education was significantly associated with right and left vmPFC_WM_ FA (right: *t* = 3.493, *p* = .0005; left: *t* = 2.442, *p* = .015) but not with FA in other ROIs (*p*'s > .08). Handedness was significantly associated with right UF FA (*t* = 2.552, *p* = .011) and right vmPFC_WM_ FA (*t* = 3.399, *p* = .0007) but not with other ROI measures (*p*'s > .06).

### 3.2 Hypotheses testing

#### 3.2.1 Associations between ROI FA asymmetry and mean neuroticism scores

**FIGURE 5:**
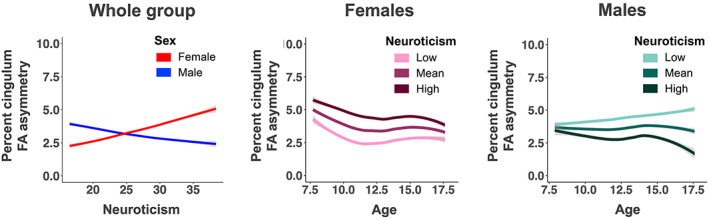
(Left) Depiction of the observed significant interaction effect between sex and neuroticism_mean_ on percent left–right cingulum FA asymmetry represented for age mean for females (red) and males (blue), with shaded ±95% confidence intervals. Higher neuroticism_mean_ scores were associated with increased left relative to right cingulum FA in females and decreased left relative to right cingulum FA in males. (Middle and right) Depiction of the associations between neuroticism_mean_, age, and cingulum FA asymmetry for, respectively, females (pink colors) and males (teal colors). Lines represent the predicted age trajectories for cingulum FA asymmetry, with shaded ±95% confidence interval bands, for different levels of neuroticism: mean neuroticism is shown in medium dark shades, while ±1 standard deviation from the mean are shown in, respectively, dark, and light shades

Results concerning our main hypotheses are presented in Table 2. As hypothesized, we found a significant neuroticism_mean_‐by‐sex interaction effect on cingulum FA asymmetry, with higher neuroticism_mean_ scores being associated with increased left relative to right cingulum FA in females and decreased left relative to right FA in males (Figure 5a, left sided plot). No significant neuroticism_mean_‐by‐sex interaction effects were observed for vmPFC_WM_ FA asymmetry, nor exploratory for UF FA asymmetry (Model 5a in Table 2). Furthermore, we did not observe any significant main effect of neuroticism_mean_ on any of the ROI FA asymmetries. Additionally, there were no statistically significant neuroticism_mean_‐by‐age or neuroticism_mean_‐by‐sex‐by‐age interaction effects (Model 5b in Table 2).

**TABLE 2:** Associations between neuroticism_mean_ and ROI FA asymmetry

		Age			Sex		Age‐by‐sex			RMS		Neuroticism		Neuroticism‐by‐sex			Neuroticism‐by‐age			Neuroticism‐by‐sex‐by‐age			
Measures	Model	edf	*F*	*p*	*t*	*p*	edf	*F*	*p*	*t*	*p*	*t*	*p*	edf	*F*	*p*	edf	*F*	*p*	edf	*F*	*p*	*R* ^2^
Cingulum FA asymmetry	5a	1.00	1.70	.193	0.18	.860	3.10	2.44	**.046**	1.93	.055	−1.70	.090	1.01	8.84	***.003***							.068
Cingulum FA asymmetry	5b	1.02	3.95	**.046**	0.26	.80	3.06	2.74	**.034**	1.76	.08	−1.65	.10	1.05	8.53	***.0035***	1.02	3.25	.07	1.88	2.55	.14	.076
VmPFC_WM_ FA asymmetry	5a	1.61	2.56	.190	0.06	.953	1.00	0.01	.920	−0.54	.587	−0.44	.662	1.00	2.28	.132							.036
VmPFC_WM_ FA asymmetry	5b	1.64	2.03	.26	0.10	.92	1	0.007	.94	0.56	.58	0.52	.61	1	2.23	.14	1.93	0.20	.79	1.00	0.05	.83	.031
UF FA asymmetry	5a	2.80	6.64	**<10** ^**−3**^	0.14	.888	1.00	7.04	**.008**	1.61	.109	−0.55	.583	1.00	0.75	.388							−.010
UF FA asymmetry	5b	2.42	3.20	**.034**	0.2	.84	1.08	3.50	.07	1.44	.15	0.51	.61	1	0.74	.39	2.27	1.65	.14	1.70	4.04	.057	.004

*Note*: Each row represents a separate GAMM model predicting ROI FA asymmetry with neuroticism_mean_ and neuroticism_mean_‐by‐sex, controlling for age, sex, age‐by‐sex and RMS. Results are based on Models 5a and 5b (Section 2.10.3). *p* values below .05 (uncorrected) are shown in bold while italic‐bold indicate *p* values that are significant after Bonferroni correction.

Abbreviations: edf, estimated degrees of freedom; FA, fractional anisotropy; GAMM, generalized additive mixed model; RMS, root mean square movement; ROI, region‐of‐interest; UF, uncinate fasciculus; vmPFC, ventromedial prefrontal cortex.

#### 3.2.2 Sex‐specific analyses

Results from the follow‐up analyses of cingulum FA asymmetry in males and females separately are represented in Table 3. For females, we observed a significant effect of neuroticism_mean_, with higher neuroticism_mean_ scores being associated with higher left relative to right cingulum FA (Figure 5). In males, the association was opposite, that is, higher neuroticism_mean_ scores were associated with lower left relative to right cingulum FA, but this association did not reach statistical significance. We did not observe significant neuroticism_mean_‐by‐age interaction effects, suggesting that the relationship between neuroticism_mean_ and cingulum FA asymmetry did not significantly change across childhood and adolescence. Visual inspection of the plots in Figure 5 displaying the association between neuroticism_mean_ and cingulum FA asymmetry across age suggested that in females the cingulum–neuroticism_mean_ relationship might already be present in the youngest part of the included age range. However, in males, an association between neuroticism_mean_ and cingulum FA asymmetry appeared to become apparent in the older part of the included age range.

**TABLE 3:** Associations of cingulum FA asymmetry and neuroticism_mean_ in females and males separately

		Age			RMS		Neuroticism		Neuroticism‐by‐age			
Cingulum FA asymmetry	Model	edf	*F*	*p*	*t*	*p*	*t*	*p*	edf	*F*	*p*	*R* ^2^
Females	6	3.10	2.73	**.032**	0.77	.440	2.45	**.015**				.066
	7	3.10	2.75	.031	0.77	.443	2.46	**.014**	1.01	0.13	.720	.066
Males	6	1.01	0.05	.828	2.45	**.015**	−1.73	.086				.067
	7	1.01	0.70	.402	2.26	**.025**	−1.84	.067	1.00	2.22	.137	.095

*Note*: Each row represents a separate GAMM model predicting cingulum FA asymmetry with neuroticism_mean_ (and age‐by‐neuroticism_mean_) controlling for age and RMS, in females or males. Results are based on Models 6 and 7 (Section 2.10.4). *p* values below .05 are shown in bold.

Abbreviations: edf, estimated degrees of freedom; FA, fractional anisotropy; GAMM, generalized additive mixed model; RMS, root mean square movement; ROI, region‐of‐interest.

### 3.3 Follow‐up analyses in females and males

#### 3.3.1 Follow‐up analyses in females

Follow‐up analyses in females showed that the association between neuroticism_mean_ and cingulum FA asymmetry remained significant (*t* = 2.535, *p* = .012), when hemispheric white matter FA asymmetry was included as an additional covariate in Model 6, suggesting that the relationship was not driven by individual differences in overall hemispheric asymmetry in FA. Moreover, the association between neuroticism_mean_ and cingulum FA asymmetry remained significant after controlling for brain volume (*t* = 2.427, *p* = .0157) or for parental education and handedness (*t* = 2.828, *p* = .005).

Next, we assessed the contribution of left and right cingulum FA to neuroticism_mean_. Neither left nor right cingulum FA was significantly associated with neuroticism_mean_ (left: *t* = − 0.248, *p* = .804; right: *t* = −1.583, *p* = .114), suggesting that the association between neuroticism_mean_ and cingulum FA asymmetry was driven by the relationship between left and right cingulum FA and not by the absolute FA values of the left or right cingulum.

Finally, to further explore the nature of our observed FA findings, we performed similar analyses as for FA (Model 6) on the cingulum AD and RD asymmetries, but no significant effects of neuroticism_mean_ on cingulum AD (*t* = 1.397, *p* = .163) or RD (*t* = −1.896, *p* = .059) asymmetry were observed.

#### 3.3.2 Post hoc analyses in males

Because the visual inspection of the plots in Figure 5 suggested that an association between neuroticism_mean_ and cingulum FA asymmetry may become apparent with age in males, we performed post hoc analyses, similar to Model 6, in, respectively, the oldest and youngest part of the age range, split by the median (12.5 years). These analyses revealed that the relationship between neuroticism_mean_ and cingulum FA asymmetry were indeed stronger in the older (*t* = −2.533, *p* = .013) than in the younger (*t* = −1.316, *p* = .191) part of the included age range.

### 3.4 Effect size maps

Effect size maps were created for males and females separately to provide further information about the distribution of the association between neuroticism_mean_ and FA across the white matter skeleton (see Supplementary results SI). The unthresholded t‐maps can be downloaded from https://neurovault.org/collections/LRMGYBHV/.

## 4 DISCUSSION

Our unique longitudinal DTI dataset enabled us to address two questions: Is neuroticism associated with interhemispheric microstructural asymmetry of fronto‐limbic white matter tracts? If so, does this association change with age in typically developing children and adolescents scanned up to 11 times? We found that higher neuroticism_mean_ scores were associated with increased FA in left cingulum relative to right cingulum in females, while higher neuroticism was associated with decreased FA in left relative to right cingulum in males. The association was most prominent in females, who also showed higher neuroticism scores than males. While neuroticism scores did not significantly change with age in males and females, there were apparent differences in the temporal trajectory of the association between neuroticism and FA asymmetry of the cingulum between males and females. In females, the association between neuroticism and cingulum FA asymmetry was already present in late childhood and remained stable during adolescence. Conversely, in males, the association became first consistently expressed during adolescence. Follow‐up analyses in females showed that the association was not driven by global hemispheric white matter FA asymmetry, handedness, or parental education. Moreover, the association appeared to be specific to the left–right asymmetry of FA, as it was not driven by absolute left or right cingulum FA values. Finally, our longitudinal analyses did not replicate our cross‐sectional baseline observation of a significant link between neuroticism and FA asymmetry of vmPFC_WM_, suggesting that the latter finding was not robust. Nor did we observe that the putative association between neuroticism and vmPFC_WM_ FA changed across time.

### 4.1 Negative emotionality trait associated with cingulum FA asymmetry

Our finding that neuroticism is associated with cingulum FA asymmetry, revealed by high temporal resolution longitudinal data, substantiate and extent our previous cross‐sectional baseline finding in the same cohort (Madsen et al., 2018). Despite age‐related increases in cingulum FA, cingulum FA asymmetry was relatively stable across the investigated age range, suggesting that cingulum FA asymmetry may be an early and stable neural correlate of neuroticism, at least in females. In general, neuroimaging studies rarely investigate regional asymmetry when investigating neuroticism and other negative emotionality‐related traits. We previously observed associations between neuroticism and cingulum FA asymmetry in an independent cohort of healthy (mainly male) adults, with higher neuroticism scores being associated with decreased left relative to right cingulum FA similar to the finding in boys of the present study (Madsen et al., 2012). However, a recent study investigating associations between negative emotionality, assessed with the Child and Adolescent Dispositions Scale (CADS) at age 10–17 years, and cingulum FA asymmetry assessed 10–15 years later in a large cohort (*n* = 410), did not find any significant associations (Lahey et al., 2020). Inconsistencies between our and Lahey's findings may in part be due to differences in personality questionnaires used, as the negative emotionality trait estimated with CADS exhibits only moderate correlations with neuroticism as well as weak, but significant correlations with conscientiousness and agreeableness (Lahey et al., 2010; Lahey et al., 2020).

Evidence supporting the importance of hemispheric asymmetry is more prominent in the electroencephalography (EEG) literature (Grimshaw & Carmel, 2014; Madsen et al., 2018; Nusslock et al., 2015), where the role of frontal cortical activity asymmetry has been investigated for more than 30 years. For example, two recent EEG studies observed that higher neuroticism scores were associated with increased left relative to right frontal alpha power during resting state (Moshirian Farahi et al., 2019) or presentation of face stimuli with a direct gaze (Uusberg et al., 2015). Of note, alpha power is assumed to be inversely correlated with cortical activity, that is, increased alpha power suggests lower neuronal activity (Allen et al., 2004; Nusslock et al., 2015). In the latter study, the neuroticism association was driven by facets related to avoidance behavior (i.e., anxiety, depression, self‐consciousness, and vulnerability), and not those related to approach behavior (i.e., angry hostility and impulsiveness). Interestingly, brain asymmetries have previously been described for approach/withdrawal behavior and emotional processing, with higher left relative to right frontal activity being associated with approach‐related traits and positive affect, while lower left relative to right frontal activity has been linked to withdrawal‐related traits and negative affect (Grimshaw & Carmel, 2014; Nusslock et al., 2015). Overall, the above suggests that cingulum asymmetry plays a role in neuroticism. It remains to be seen whether associations are driven by specific subcomponents of neuroticism. Furthermore, future studies should establish whether cingulum FA asymmetry plays a role in other negative emotionality traits.

### 4.2 Clinical implications of the association between neuroticism and regional brain asymmetry

High neuroticism scores have been associated with a wide range of psychiatric disorders (Hettema et al., 2006; Kendler & Myers, 2010; Tully et al., 2015). Genes linked to neuroticism are co‐expressed with many psychiatric disorders, including mood and anxiety disorders (Anttila et al., 2018; De Jager et al., 2021; Kendler & Myers, 2010; Luciano et al., 2018). However, overlapping brain structural correlates between neuroticism and mood and anxiety disorders are lacking. We suggest that structural and functional asymmetries of fronto‐limbic brain regions and fiber tracts may represent a potential marker of risk, as such asymmetries have also been related to mood and anxiety disorders (Chanen et al., 2008; Kim et al., 2006; Nusslock et al., 2015). This assumption is supported by findings showing that healthy adolescents, who were at high risk for depression, had lower FA in the left cingulum (Huang et al., 2011), and that young adults with decreased left relative to right frontal resting EEG activity had greater probability for having a depressive episode during a 3‐year follow‐up period (Nusslock et al., 2011). Furthermore, reduced asymmetry in frontal alpha power in resting‐state EEG has been linked to major depression, either due to a reduced left frontal or an increased right frontal activity (Thibodeau et al., 2006). Finally, FA asymmetry of the cingulum has also been linked to the stress hormone cortisol. Madsen et al. (2012) showed associations between higher cortisol awakening response, higher neuroticism scores, and decreased left relative to right cingulum FA in a predominantly male cohort of healthy adults. Like neuroticism, higher cortisol awakening response is a known risk factor for developing affective disorders (Adam et al., 2010). Our present findings indicate that, despite ongoing age‐related increases in cingulum FA, cingulum FA asymmetry is relatively stable across late childhood and adolescence. Further, since the association with neuroticism was stable for females across this developmental period, it seems unlikely that cingulum FA symmetry per se is linked to the observed differences in the prevalence of anxiety and mood disorders between females and males during adolescence. Taken together, asymmetric patterns might already be present in at risk individuals and remain in individuals with manifest disorder. We speculate that asymmetry in cingulum FA may be a brain structural marker for a predisposition or risk for developing affective disorders. Future (prospective) studies in healthy and clinical cohorts are needed to clarify whether asymmetry within the frontal‐limbic circuitry may predispose a risk of developing affective disorders.

### 4.3 Sex differences in the association between neuroticism and cingulum FA asymmetry

We observed opposite relationships between neuroticism and cingulum FA asymmetry for females and males. Higher neuroticism scores were associated with increased left relative to right cingulum FA in females and decreased left relative to right cingulum FA asymmetry in males, substantiating our previous cross‐sectional findings in the same cohort (Madsen et al., 2018). Since females and males did not significantly differ in cingulum FA asymmetry, the observed sex differences in neuroticism‐cingulum associations are likely not driven by structural sex differences in cingulum FA per se. Opposite sex effects have also been observed in the relationship between neuroticism and grey matter volume and cortical thickness of the subgenual anterior cingulate cortex in adolescents aged 16–17 years, with females showing positive and males negative correlations (Blankstein et al., 2009). Further, a cross‐sectional study in adults aged 19–80 years reported that higher neuroticism was associated with thicker anterior cingulate cortex in females and thinner anterior cingulate cortex in males with increasing age (Sweeney et al., 2019). Notably, the anterior cingulate cortex projects to and receives fibers from the cingulum. Overall, these findings suggest that there are sex differences in the associations between neuroticism and the cingulum and related cortical regions. However, the mechanisms underlying these associations are still unknown, and possible explanations may be linked to biological factors such as sex hormones (prenatal and pubertal), stress hormones and/or genetic differences, as well as to psychological and/or socio‐cultural factors. A clear differentiation between biological and socio‐cultural factors is challenging due to their interdependence. In support of a biological interpretation, cross‐cultural studies consistently show that women exhibit higher neuroticism scores than men (Mac Giolla & Kajonius, 2019; Schmitt et al., 2008), regardless of age (Costa et al., 2001). Surprisingly, however, sex differences in neuroticism are larger in more gender equal countries (Mac Giolla & Kajonius, 2019; Schmitt et al., 2008) suggesting that socio‐cultural factors may also play a role.

Our longitudinal study design allowed us to track changes in the neural correlates of neuroticism across time. In females, the relationship between neuroticism and cingulum FA asymmetry was already present in the youngest part of the included age range and appeared to be stable across childhood and adolescence. This suggests that the relationship arose earlier in life, that is, before late childhood, and we speculate that it might be innate. Indeed, (leftward) cingulum FA asymmetry appears to be present already in infants and remain throughout childhood, adolescence, and early adulthood (Cohen et al., 2016). In line with this, we observed that (leftward) cingulum FA asymmetry did not significantly change with age, though FA increased significantly in both the left and the right cingulum. Furthermore, both neuroticism (Sanchez‐Roige et al., 2018; van den Berg et al., 2014) and cingulum DTI measures (Budisavljevic et al., 2016; Peper et al., 2008; Shen et al., 2016; Vuoksimaa et al., 2017; Zhao et al., 2019) are moderately heritable, suggesting that at least part of the observed associations between neuroticism and cingulum FA asymmetry may be linked to genetic differences. Finally, since increased prenatal stress has been linked to increased fearfulness in toddlers (Bergman et al., 2007) and altered functional brain asymmetry in both rodents and healthy children (Alonso et al., 1991; Jones et al., 2011), as well as lower FA in the left cingulum in young adults (Marečková et al., 2019), we speculate that prenatal stress might also play a role in the observed associations between neuroticism and cingulum FA asymmetry.

Although we were unable to find statistically significant age‐related changes in the link between neuroticism and cingulum FA asymmetry in males, visual inspection of the age trajectories of this relationship suggested that the association may first become apparent during adolescence. Furthermore, our post hoc analyses of the youngest and oldest male participants suggested that the neuroticism‐cingulum FA asymmetry relationship was only apparent in the older age range. Speculatively, neuroticism‐cingulum FA asymmetry associations may be less stable in males and may be influenced by moderating factors during adolescence. Interestingly, higher endogenous testosterone levels have been associated with lower neuroticism levels in adolescent and young adult males (Schutter et al., 2017). Furthermore, in the same study, lower neuroticism levels were linked to larger cerebellar volume, and this relationship appeared to be mediated by endogenous testosterone levels. Moreover, in a recent study in adolescent males, using transcranial Doppler ultrasonography, pubertal testosterone levels affected the extent of asymmetric brain activation during a mental rotation task and a chimeric face task. Intriguingly, the direction of the association depended on the levels of prenatal testosterone exposure, suggesting that prenatal and pubertal testosterone levels might affect functional brain asymmetries in a complex manner (Beking et al., 2018). As pubertal testosterone may affect functional brain asymmetries in adolescent males, we speculate that pubertal testosterone levels may affect the relationship between neuroticism and cingulum FA asymmetry in males during adolescence. Future studies are needed to clarify whether pubertal testosterone affects the relationship between neuroticism and cingulum FA asymmetry in males during adolescence.

### 4.4 Strength and limitations

The current study has several strengths and limitations. A major strength of this study is its longitudinal design with high temporal resolution and up to 11 MRI scans per individual, optimized to investigate changes in brain–behavior relationships across late childhood and adolescence. Furthermore, since the intraindividual reliability and agreement of the longitudinal neuroticism scores were considered good, and the longitudinal neuroticism scores did not significantly change with age in the investigated age range, we were able to use the mean neuroticism score, which allowed us to capitalize all the longitudinal MRI assessments as well as include participants with only one neuroticism assessment, thereby enhancing statistical power. Nevertheless, using mean neuroticism scores instead of longitudinal neuroticism scores might have prevented us from finding subtle changes in the relationship between neuroticism and associated white matter correlates. However, since our intra‐individual reliability measure of neuroticism_long_ (ICC = 0.74) was within the range of previously reported 1‐month test–retest reliability estimates (0.64–0.82) of J‐EPQ neuroticism scores in 11–14‐year‐olds (Eysenck & Eysenck, 1975; Nyborg et al., 1982), we speculate that using neuroticm_mean_ instead of neuroticism_long_ might have decreased the variability associated with everyday fluctuations. Additionally, we specifically covered the period of late childhood and adolescence (7–18 years), allowing us to trace changes in the peri‐pubertal developmental period, where sex differences in neuroticism have been reported to emerge (De Bolle et al., 2015). However, since the cingulum show protracted maturation into early adulthood (Lebel et al., 2008), future studies should consider including a broader age range encompassing early childhood and adulthood. Finally, our sample size consisted of more females (*n* = 47) than males (*n* = 29), which might have prevented us from finding any significant neuroticism associations with cingulum FA asymmetry and neuroticism in males as well as significant sex differences in the association between neuroticism and cingulum FA asymmetry over age.

## 5 CONCLUSIONS

We sampled structural brain development with MRI at high temporal resolution, covering late childhood and adolescence. Our unique longitudinal data revealed robust associations between trait neuroticism scores and cingulum FA asymmetry in typically developing children and adolescents aged 7–18 years, with opposite effects in females and males. In females, higher neuroticism was associated with increased left relative to right cingulum FA, while in males, higher neuroticism was associated with decreased left relative to right cingulum FA. The association appeared to be stable across late childhood and adolescence in females, and we speculate that this association might be innate. In males, however, the association appeared to be stronger in the higher end of the investigated age range, and we hypothesize that increasing pubertal testosterone levels might play a role in this. Future studies should cover a larger age span to elucidate when the relationship between neuroticism and cingulum FA asymmetry arises and to clarify the possible role of sex hormones, as well as genetic variation and prenatal stress exposure.

## AUTHOR CONTRIBUTIONS

**Anna Plachti**: Conceptualization, Software, Formal analysis, Data curation, Writing ‐ original draft, review and editing, Visualization. **William F. C. Baaré**: Conceptualization, Methodology, Software, Resources, Data curation, Writing ‐ original draft, review and editing, Supervision, Project administration, Funding acquisition. **Louise Baruël Johansen**: Software, Formal analysis, Data curation, Writing ‐ review and editing, Visualization. **Wesley K. Thompson**: Software, Writing ‐ review and editing. **Hartwig R. Siebner**: Resources, Writing ‐ review and editing, Funding acquisition. **Kathrine Skak Madsen**: Conceptualization, Methodology, Software, Investigation, Resources, Data curation, Writing ‐ original draft, review and editing, Visualization, Supervision, Project administration, Funding acquisition.

## FUNDING INFORMATION

This work was supported by the Danish council of Independent Research|Medical Sciences (grant numbers 09‐060166 and 0602‐02099B), the Lundbeck Foundation (grant number R32‐A3161), the Lundbeck Foundation Center of Excellence grant to The Center for Integrated Molecular Brain Imaging, and EU Horizon 2020 research and innovation program grant for the Lifebrain project (grant agreement number 732592). Hartwig R. Siebner holds a 5‐year professorship in precision medicine at the Faculty of Health Sciences and Medicine, University of Copenhagen, which is sponsored by the Lundbeck Foundation (grant number R186‐2015‐2138).

## CONFLICT OF INTEREST

Hartwig R. Siebner has received honoraria as speaker from Sanofi Genzyme, Denmark and Novartis, Denmark, as consultant from Sanofi Genzyme, Denmark, Lophora, Denmark, and Lundbeck AS, Denmark, and as editor‐in‐chief (Neuroimage Clinical) and senior editor (NeuroImage) from Elsevier Publishers, Amsterdam, The Netherlands. He has received royalties as book editor from Springer Publishers, Stuttgart, Germany and from Gyldendal Publishers, Copenhagen, Denmark. The authors declare no potential conflict of interest.

## Supporting information


Appendix S1: Supporting Information

## Data Availability

Individual level data for the HUBU Study is confidential. However, the full unthresholded t‐maps have been uploaded to NeuroVault.org and are available at https://neurovault.org/collections/LRMGYBHV/.
