# Supplementary material for: Stability of associations between neuroticism and microstructural asymmetry of the cingulum during late childhood and adolescence: Insights from a longitudinal study with up to 11 waves
Source: Hum Brain Mapp. 2022 Nov 25;44(4):1548–64. doi: 10.1002/hbm.26157 (PMC9921236; doi:10.1002/hbm.26157)
Supplement: Supplementary file 1 — Appendix S1: Supporting Information [file HBM-44-1548-s001.docx]

**Supplementary Material**

**Supplementary results**

**Effect size maps**

The effect size maps display the t-values where FA was positively (red-yellow colors) or negatively (blue-light blue colors) associated with neuroticism_mean_, controlling for age and RMS. The full unthresholded t-maps can be downloaded from https://neurovault.org/collections/LRMGYBHV/. In the description below, the numbers between brackets represent the MNI Y coordinate depicted in Supplementary Figure 1.

In females, neuroticism_mean_ was positively and negatively associated with FA (3.3 ≤ t ≤ -3.3, p < 0.001, uncorrected), in respectively, 1.17% and 4.12% of all skeleton voxels, excluding voxels for which the GAMM model did not converge (0.44%). Higher neuroticism_mean_ scores were associated with higher FA in several clusters across the white matter skeleton, including the splenium of corpus callosum (-43) and the temporal part of the right superior longitudinal fasciculus (-50), as well as the white matter underlying the left anterior parahippocampal gyrus (2), and left supramarginal gyrus (-50). Furthermore, lower neuroticism_mean_ was associated with higher FA in multiple white matter regions, including the left inferior fronto-occipital fasciculus (33), right UF (8), left inferior longitudinal fasciculus (-9), and bilateral anterior thalamic radiation (-9) as well as the white matter underlying the middle and superior frontal gyrus (24), right temporal pole (8), right inferior precentral gyrus (-3), left subcentral gyrus (-3, -6), left superior temporal gyrus (-16), right supramarginal gyrus (-36), and right middle temporal gyrus (-40).

In males, we found that neuroticism_mean_ was positively and negatively associated with FA in, respectively, 4.2% and 2.3% of all skeleton voxels, excluding voxels for which the GAMM model did not converge (0.34%). Higher neuroticism_mean_ was associated with higher FA in multiple clusters across the white matter skeleton, including the right UF (43, 2), genu of corpus callosum (26), bilateral inferior fronto-occipital fasciculus (43, -49, -73), right corticospinal tract (-30), bilateral superior longitudinal fasciculus (-30, -49), left posterior corona radiata (-35), bilateral posterior thalamic radiation (-49, -51), bilateral inferior longitudinal fasciculus (-49, -73), forceps major (-73) as well as the white matter underlying the left superior frontal gyrus (43), bilateral inferior frontal gyrus (26, -3), right supplementary motor area (-3), bilateral postcentral gyrus (-18), and right superior parietal lobule (-81). Further, lower neuroticism_mean_ scores were associated with higher FA in right UF/inferior longitudinal fasciculus (2) and right forceps major (-81) as well as the white matter underlying the left superior frontal gyrus (37), left posterior frontal opercular cortex (2), and left precuneus (-49).

Additional t- and F-maps of the association of FA with neuroticism_mean_-by-age for the whole group and females and males separately have also been uploaded to NeuroVault.


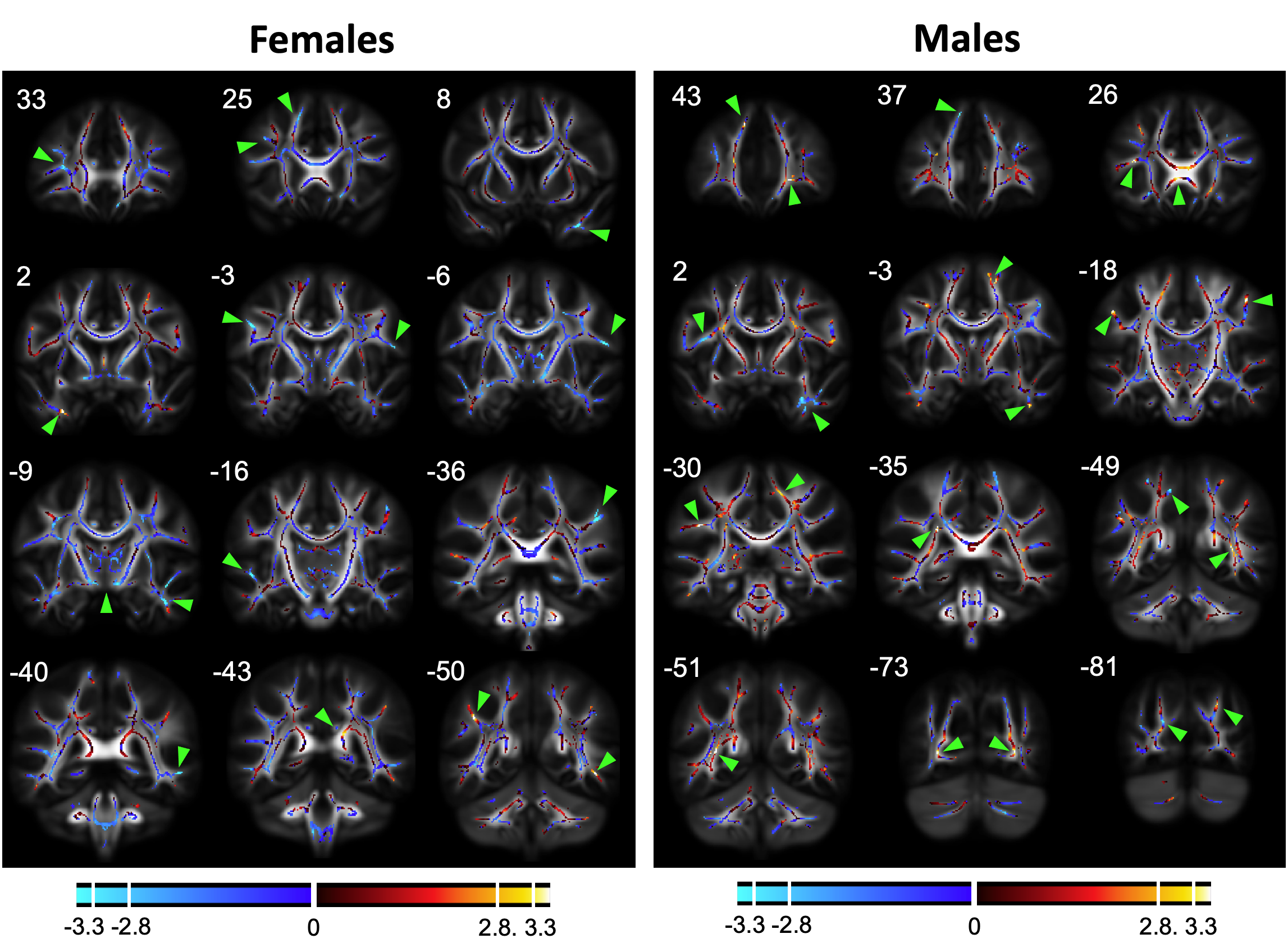


**Supplementary Figure 1**. Effect size map displaying the association between neuroticism_mean_ and FA across the white matter skeleton, corrected for age and RMS. The effect size map displays the images with the highest t-values and largest clusters, based on visual inspection, with the main clusters pointed out using green arrows. Voxels, where higher neuroticism_mean_ was associated with higher FA, are depicted in warm colors ranging from red to yellow, while cold colors ranging from dark blue to light blue depict where higher neuroticism_mean_ was associated with lower FA. The t-values in the color bar correspond to t = ± 3.3, p = 0.001 and t = ± 2.8, p = 0.005 (df = 366 for females, df = 226 for males, two-tailed, uncorrected). The MNI Y coordinates for the coronal slices are given above each image. Images are shown according to neurological convention, with the left hemisphere depicted in the left side.
